# Screening for infectious and neglected tropical diseases among newly arrived migrants from Africa and Asia: a retrospective study from Verona province, Italy

**DOI:** 10.1186/s41182-025-00796-4

**Published:** 2025-08-28

**Authors:** Tamara Ursini, Lucia Bonato, Amina Zaffagnini, Cristina Mazzi, Paolo Cattaneo, Elena Salvador, Lucia Moro, Federico Gobbi, Dora Buonfrate

**Affiliations:** 1 Department of Infectious - Tropical Diseases and Microbiology, IRCCS Sacro Cuore Don Calabria Hospital, Negrar Di Valpolicella, Verona, Italy; 2 Infectious Disease Unit, Mater Salutis Hospital – aulss9, Legnago, Verona, Italy; 3 Department of Clinical and Experimental Sciences, University of Brescia, Brescia, Italy

**Keywords:** Migrants, Screening, Tuberculosis, Helminths, Schistosomiasis, Strongyloidiasis

## Abstract

**Background:**

Migration to Europe has increased in recent years, with Italy serving as a major entry point. Ensuring adequate healthcare for newly arrived migrants includes the prevention and management of infectious diseases. This study aimed to estimate the prevalence of selected infections among migrants in northern Italy.

**Methods:**

We conducted a retrospective cross-sectional study including newly arrived migrants screened at the Department of Infectious—Tropical Diseases and Microbiology (DITM) of the IRCCS Sacro Cuore Don Calabria Hospital, Negrar di Valpolicella (Verona, Italy) between January 2023 and May 2024.

Asylum seekers and undocumented migrants aged ≥ 14 years who had arrived within the previous six months from Africa or Asia were screened for tuberculosis (TB), HIV, hepatitis B (HBV), hepatitis C (HCV), syphilis, strongyloidiasis, schistosomiasis, other intestinal helminthic infections, and filariasis. Diagnostic methods comprised serological, microscopic, molecular, and imaging techniques, applied as appropriate.

**Results:**

Among the 674 migrants screened (median age: 25 years; 86.4% male), TB infection was detected in 25.4%, and 2.9% were diagnosed with TB disease. HIV prevalence was 1.5%, primarily among individuals from sub-Saharan Africa. Chronic HBV infection was identified in 6.1% of participants, while 55.1% were seronegative —i.e., negative for HBsAg, anti-HBs, and anti-HBc IgG— and thus eligible for vaccination. Helminthic infections were found in 12.3%, mainly strongyloidiasis and schistosomiasis. Eosinophilia was present in 18.3% and was significantly associated with schistosomiasis, strongyloidiasis, and hookworm infection (all p < 0.05).

**Conclusions:**

These findings underscore the consistent burden of infectious diseases among migrant populations and support the implementation of geographically tailored screening programs to improve early detection and public health outcomes.

## Background

Over the last decade, international migration has increased across all United Nations (UN) regions, with the most significant growth observed in Europe and Asia [1]. In Europe alone, the number of international migrants rose by nearly 16%, from approximately 75 million in 2010 to 87 million in 2020 [1].

Italy has experienced fluctuating migration flows during this period. Between 2014 and 2017, the country saw a sharp increase in sea arrivals, often reported as “refugee crisis” in political and media discourse [2]. The peak was recorded in 2016, with 181,436 migrants arriving by sea. However, from 2018 onward, arrivals declined significantly before rebounding in 2020. In both 2022 and 2023, over 100,000 migrants reached Italy, driven mainly by increased flows from North and Central Africa, as well as a steady rise in arrivals from Asian countries such as Bangladesh and Pakistan [2].

The health implications of migration have been a focus of public health policies within the European Union/European Economic Area (EU/EEA). In 2018, the European Centre for Disease Prevention and Control (ECDC) published Public Health Guidance on screening and vaccination for infectious diseases in newly arrived migrants, recommending screening for tuberculosis (TB), tuberculosis infection (TBI), human immunodeficiency virus (HIV), hepatitis B (HBV), hepatitis C (HCV), schistosomiasis, and strongyloidiasis [3]. Early detection and treatment are essential both for individual health—preventing disease progression and late complications—and for public health, by reducing transmission in host countries [3].

Several infectious diseases relevant to migrant populations can have severe long-term consequences if left untreated. Communicable diseases such as TB, viral hepatitis, HIV, and syphilis pose transmission risks, while non-communicable infections like strongyloidiasis, schistosomiasis, and filariasis may lead to chronic conditions associated with significant morbidity [4, 5]. For instance, schistosomiasis can lead to chronic urogenital, hepato-intestinal, and central nervous system complications, while *Strongyloides stercoralis* infection may cause disseminated disease or fatal hyperinfection in immunosuppressed patients [6]. Moreover, certain infections carry a risk of local transmission in the EU/EEA, either through organ transplantation (e.g. strongyloidiasis) or via environmental conditions that support the intermediate host, as seen in recent autochthonous cases of urinary schistosomiasis in Corsica, France [6, 7]. Importantly, many of these conditions are treatable with short, well-tolerated outpatient regimens, reinforcing the value of systematic screening [5].

Screening protocols for infectious diseases among newly arrived migrants are crucial for identifying and managing infections that, while uncommon in the host country, may have significant epidemiological and clinical implications, and for ensuring their appropriate management in routine clinical practice A previous study conducted at the Department of Infectious—Tropical Diseases and Microbiology (DITM) assessed the prevalence of a series of infectious diseases among asylum seekers temporarily residing in Verona province between April 2014 and June 2015 [8]. The findings highlighted the importance of including neglected helminthic infections in screening strategies, given their prevalence and the favorable safety profile of available treatments [8].

Tracking changes in the prevalence of infectious diseases among migrants from different regions over time is essential for refining and validating current screening strategies. This study aims to estimate the prevalence of a range of infectious diseases, both communicable and non-communicable, in a cohort of recently arrived asylum seekers and undocumented migrants in Italy.

The study focused on infections and diseases of public health relevance, including TB infection (TBI), HIV infection, viral hepatitis (HBV and HCV), syphilis, strongyloidiasis, schistosomiasis, other intestinal helminthic infections, and filariasis. Additionally, as a secondary objective, we will explore the predictive role of eosinophilia in the context of the screening, analyzing its association with helminthic infections.

## Methods

### Study design

This was a retrospective observational cross-sectional study analyzing data from infectious disease screening activities conducted at the Department of Infectious—Tropical Diseases and Microbiology (DITM) of the IRCCS Sacro Cuore Don Calabria Hospital, Negrar di Valpolicella (Verona, Italy) from January 2023 to May 2024. Given its retrospective nature, the study did not involve follow-up of participants but rather aimed to describe the prevalence of infectious diseases among recently arrived migrants.

### Study population and setting

The study population included asylum seekers and undocumented migrants aged ≥ 14 years who had arrived within the past six months from Africa and Asia and attended the dedicated outpatient service at DITM for medical screening. Individuals were either referred by local reception centers or presented spontaneously at the outpatient service. Access to screening was granted regardless of the presence or absence of symptoms. Demographic data were collected from official documents issued by the local prefectures where the individuals had applied for asylum. For undocumented migrants, available personal data were obtained from any documents they had at the time of screening.

### Study procedures

All migrants underwent a general medical examination. In addition to a full blood cell count (FBC), diagnostic tests for specific infections were proposed.

- TB screening was conducted using the QuantiFERON-TB Gold In-Tube (QFT-GIT) assay (LIAISON^®^ QuantiFERON^®^-TB Gold Plus, DiaSorin) and chest X-rays.
- HIV screening was performed with an indirect immunoenzymatic assay (HIV Combo V2 Immunoassay System, Biorad), and a Western blot (INNO-LIA, Fujirebio Diagnostics) was used as a confirmatory test.
- HBV screening was conducted using the following assays: a qualitative immunoenzymatic assay for HBV core antibody, a quantitative chemiluminescence immunoassay (CLIA) for HBV surface antibody, and a qualitative CLIA for HBV surface antigen (Access HBcAb, Access HBsAb, Access HBsAg, Beckman Coulter).
- Anti-HCV antibodies were detected using chemiluminescent immunoassay (CLIA) (Access anti-HCV, Beckman Coulter).
- Syphilis screening was conducted using CLIA for *Treponema pallidum*, with confirmation by the *T. pallidum* haemagglutination assay (TPHA) (LIAISON® Treponema Screen, DiaSorin) and the Rapid Plasma Reagin (RPR) test (Mascia Brunelli S.p.A.).
- Helminthic infections were assessed using the following methods:
  o Stool microscopy for ova and parasites after formol-ether concentration.
  o Urine microscopy after micropore filtration for *Schistosoma haematobium* (only for individuals from sub-Saharan Africa).
  o Serology for *Schistosoma* spp*.* (Schistosoma mansoni ELISA kit, Bordier Affinity Products SA) and/or immunochromatographic test kit ICT Bordier Affinity Products SA.
  o Additionally, urine from selected patients was tested for *Schistosoma* spp*.* using in-house PCR.
  o *Strongyloides stercoralis* infection was screened by serology (in-house immunofluorescence assay [IFAT] and/or ELISA kit, Bordier Affinity Products SA). Stools from subjects with positive serology were also tested by agar plate culture (APC).
  o *Filaria* spp. infection was screened in migrants from sub-Saharan Africa using serology (Acanthoecheilonema viteae IgG ELISA kit, Bordier Affinity Products SA). All patients with positive *Filaria* serology underwent additional testing for daytime and/or nighttime microfilaremia.
  o Additionally, stool samples from selected patients, based on clinical evaluation, were tested by in-house PCR amplification for the following parasites: *Strongyloides* spp*.*, *Schistosoma* spp*.*, *Hymenolepis nana*, *Dientamoeba* spp*.*, *Giardia intestinalis*, *Blastocystis* spp*.*, *Entamoeba histolytica*, *Entamoeba dispar*, *Cryptosporidium* spp*.*, *Ascaris lumbricoides*, *Ancylostoma duodenale*, *Necator americanus*, and *Trichuris trichiura*.
- Results were recorded anonymously in an Excel database.

Post-screening management varied according to the condition diagnosed. Although therapeutic management was not the focus of the present study, all individuals with a confirmed diagnosis received appropriate care in line with clinical standards. Individuals diagnosed with HIV or chronic viral hepatitis were referred to specialized infectious disease centers for further evaluation and care. Most cases of syphilis, tuberculosis, and helminthic infections (e.g., strongyloidiasis, schistosomiasis) were managed either through outpatient follow-up or hospitalization at our center, depending on clinical severity. In the case of helminthic infections, individuals with positive serology for *Strongyloides stercoralis* or *Schistosoma* spp. were treated regardless of parasitological confirmation, in accordance with international recommendations [5].

### Variables

Key study variables included demographic data, clinical findings, and laboratory results.

- Eosinophilia was defined as an absolute eosinophil count ≥ 400 cells/µL.
- Positivity for HIV, HBV, HCV, and syphilis was assessed via serological testing; chronic HBV infection was identified by HBsAg positivity, and anti-HBs and anti-HBc IgG were also tested to assess immune status.
- TBI was diagnosed in individuals with a positive QFT-GIT test, who showed no signs or symptoms of TB disease and had a negative chest X-ray.
- TB disease was defined based on imaging findings (e.g., chest X-ray for pulmonary TB and other imaging modalities for extrapulmonary TB) compatible with clinical tuberculosis, regardless of the presence or absence of symptoms. Subclinical and symptomatic TB were not distinguished and were both included under this definition.
- Previously treated TB was defined as a history of disease based on self-reported prior treatment and/or radiological findings suggestive of past TB sequelae, such as fibrotic lesions or calcifications, in the absence of symptoms.
- Strongyloidiasis was defined by a positive serology and at least one positive fecal test.
- Schistosomiasis was defined by a positive serology and at least one positive fecal or urinary test.
- Filariasis was defined by a positive serology and the presence of microfilaremia.
- Intestinal helminthic infections were defined by the detection of parasites in fecal samples, either through stool microscopy or PCR-based techniques.

Missing data were recorded and analyzed, with descriptive statistics used to assess their potential impact on the results.

### Sample size

A convenient sample of all eligible records of migrants screened between January 2023 and May 2024 were considered for analysis.

### Statistical analysis

Diagnostic test results were categorized as binary (positive/negative), while for continuous variables, median and interquartile ranges (IQR) were reported. Frequencies and percentages were reported for categorical variables. The primary outcome was the prevalence of the infections of interest. Prevalence was expressed as the frequency of positive tests over the total number of cases tested, and reported with 95% confidence intervals calculated using the Clopper and Pearson formula. Comparisons between proportions were made using Fisher exact test or Chi square test. A multivariable Firth logistic regression model was used to assess potential risk factors of parasite infections (*S*. *stercoralis*, *T. trichiura*, hookworm, *Schistosoma* spp. and *Filaria* spp.) for eosinophilia, separately for Asia and sub-Saharan Africa, and adjusted for age and sex. Estimates were reported as odds ratios (OR) and 95% confidence intervals (CI). P-values lower than 0.05 were considered significant. Analyses were performed using R software version 4.4.2.

### Ethical considerations

The study was approved by the local ethics committee (Comitato Etico per la Sperimentazione Clinica delle Province di Verona e Rovigo, protocol number 29, July 2, 2024). Of note, all individuals accessing our laboratory service are routinely asked to sign a general informed consent for the use of pseudo-anonymised clinical data and biological samples for research purposes under the protocol of the institutional Tropica Biobank.

## Results

**Fig. 1:**
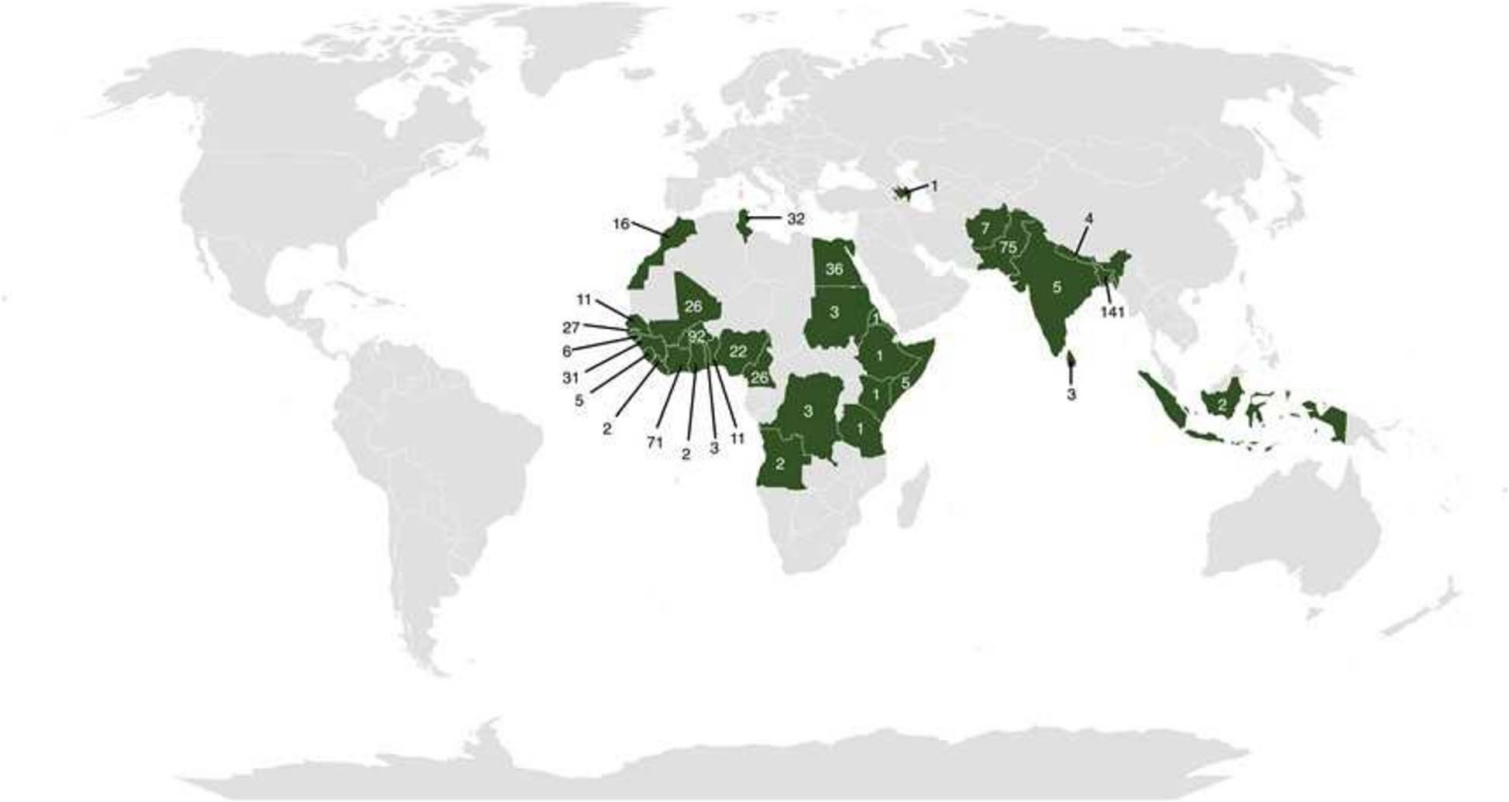
Geographic distribution of study participants: countries of origin highlighted in green with participant numbers. Afghanistan (7, 1.04%), Angola (2, 0.30%), Azerbaijan (1, 0.15%), Bangladesh (141, 20.92%), Benin (11, 1.63%), Burkina Faso (92, 13.65%), Cameroon (26, 3.86%), Côte d'Ivoire (71, 10.53%), Democratic Republic of Congo (3, 0.45%), Egypt (36, 5.34%), Eritrea (1, 0.15%), Ethiopia (1, 0.15%), Gambia (27, 4.01%), Ghana (2, 0.30%), Guinea (31, 4.60%), Guinea-Bissau (6, 0.89%), India (5, 0.74%), Indonesia (2, 0.30%), Kenya (1, 0.15%), Liberia (2, 0.30%), Mali (26, 3.86%), Morocco (16, 2.37%), Nepal (4, 0.59%), Nigeria (22, 3.26%), Pakistan (75, 11.13%), Senegal (11, 1.63%), Sierra Leone (5, 0.74%), Somalia (5, 0.74%), Sri Lanka (3, 0.45%), Sudan (3, 0.45%), Tanzania (1, 0.15%), Togo (3, 0.45%), Tunisia (32, 4.75%)

Overall, 674 individuals were screened and included in the analysis. The median age was 25 years (IQR 20–31) and 86.4% (n = 582) was male. As regards the geographic area of origin, 52.2% (n = 352) came from sub-Saharan Africa, 35.3% (n = 238) originated from Asia, and 12.5% (n = 84) from North Africa. Figure 1 shows the number of participants from each country of origin.

The median time spent in Italy was 3 months (IQR 1—5).

Demographic characteristics of participants by macro-area are reported in Table 1.

**Table 1:** Demographic Characteristics of Study Participants

	Region			
	Overall	Sub-Saharan Africa	Asia	North Africa
	N = 674	N = 352	N = 238	N = 84
Median age in years (IQR)	25 (20–31)	23 (19–29)	27 (23–34)	24 (18–32)
Male, n (%)	582 (86.4%)	284 (80.9%)	223 (93.7%)	75 (89.3)
Median number of months in country (IQR)	3 (1–5)	2 (1–5)	3 (2–5)	2 (1–6)

### Viral infections

Of the 672 participants screened for HIV, 10 (1.5%) tested positive. Nine were from sub-Saharan Africa (9/10; 90%) and one from Bangladesh (1/10; 10%).

A total of 673 migrants were screened for HBV infection. Of these, 371 (55.1%) had negative serology and were therefore eligible for vaccination, including 124 out of 352 from sub-Saharan Africa (35.2% of individuals from this region), 180 out of 237 from Asia (75.9%), and 67 out of 84 from North Africa (79.8%).

Forty-one individuals (6.1%) tested positive for HBsAg, indicating chronic HBV infection. The majority were from sub-Saharan Africa (31/352; 8.8% of migrants from this region), followed by Asia (9/237; 3.8%) and North Africa (1/84; 1.2%).

In addition, 261 individuals (38.8%) tested positive for anti-HBs and/or anti-HBc IgG, indicating past exposure or immunity to HBV, and therefore were not eligible for vaccination.

Of the 658 migrants tested for HCV, five (0.8%) tested positive. One was from sub-Saharan Africa (1/339; 0.3% of individuals from this region), three from Asia (3/235; 1.3%)—all from Pakistan—and one from North Africa (1/84; 1.2%). Among those with positive serology, HCV-RNA was detectable in one individual (from Pakistan), undetectable in three (two from Pakistan and one from sub-Saharan Africa), and unavailable for the participant from North Africa (Egypt).

### Bacterial infections

Out of 635 individuals tested for syphilis, 13 (2.0%) were positive. Of these, 12 were from sub-Saharan Africa (12/341; 3.5% of migrants from this region), one from Asia (1/213; 0.5%), and none from North Africa (0/81; 0.0%).

A total of 653 migrants were screened for tuberculosis using QFT-GIT: 188 (28.8%) tested positive, 460 (70.4%) negative, and five (0.8%) had indeterminate results. Among the 188 QFT-positive individuals, 170 underwent chest X-ray, which revealed 160 cases (24.5%) of tuberculosis infection (TBI). Two individuals (0.3%) had previously treated TB, and eight (1.2%) were diagnosed with TB disease (Table 2). For the purposes of this study, we did not differentiate between subclinical and symptomatic presentations of TB disease.

**Table 2:** Tuberculosis screening results

	Region				p-value*
	Overall	Sub-Saharan Africa	Asia	North Africa	
					< 0.001
Negative n (%)	460 (70.4%)	207 (61.2%)	181 (78.3%)	72 (85.7%)	
TBI n (%)	160 (24.5%)	111 (32.8%)	43 (18.6%)	6 (7.1%)	
Previously treated TB n (%)	2 (0.3%)	1 (0.3%)	0 (0.0%)	1 (1.2%)	
PTB n (%)	5 (0.8%)	2 (0.6%)	3 (1.3%)	0	
EPTB n (%)	3 (0.5%)	2 (0.6%)	1 (0.4%)	0	
Missing n	21	14	7	0	

^*^ Fisher’s exact test

TBI: Tuberculosis infection; PTB: Pulmonary TB; EPTB: Extrapulmonary TB

### Helminthic infections

At least one stool sample for microscopy was provided by 618 (91.7%) subjects, of whom 333 out of 352 (94.6%) from sub-Saharan Africa, 213 out of 238 (89.5%) from Asia, and 72 out of 84 (85.7%) from North Africa. A significant association was detected between geographical region and positivity at stool microscopy (chi-squared test, p-value < 0.001).

Of the 642 samples examined by either stool microscopy or PCR, 79 (12.3%) were positive for at least one helminth. Most positive individuals were from sub-Saharan Africa (62, 78.5%), followed by Asia (14, 17.7%) and North Africa (3, 3.8%). Data concerning the results of stool microscopy are summarized in Table 3.

**Table 3:** Stool microscopy results

	Region			
	Overall	Sub-Saharan Africa	Asia	North Africa
Individuals screened by stool microscopy	618	333	213	72
Positive for *S. stercoralis* larvae n (%)	3 (0.5%)	1 (0.3%)	2 (0.9%)	0
Positive for *S. mansoni* eggs n (%)	28 (4.5%)	28 (8.4%)	-	0
Positive for hookworm^1^ eggs n (%)	12 (1.9%)	6 (1.8%)	6 (2.8%)	0
Positive for *A. lumbricoides* eggs n (%)	1 (0.2%)	1 (0.3%)	0	0
Positive for *T. trichiura* eggs n (%)	6 (1.0%)	0	6 (2.8%)	0
Positive for other parasites^2^ n (%)	244 (39.5%)	163 (48.9%)	57 (26.8%)	24 (33.3%)

^1^ Includes *Ancylostoma duodenale* and *Necator americanus*

^2^ Reporting is focused on helminths determined to be clinically relevant, such as soil-transmitted helminths. Some other parasites, both helminths and protozoa, might not have a clinical relevance and are included in the other parasites group (e.g. *Hymenolepis nana* and *Endolimax nana*)

*Strongyloides stercoralis* was detected by a number of tests, including serology, APC and PCR. Table 4 displays the results of all screening tests used, per geographical origin. While the number of positive *S. stercoralis* serology tests was significantly larger among individuals from Asia compared to the other geographical areas, figures did not significantly differ per geographical origin when considering all stool tests. Overall, 9 out of 673 individuals were positive to at least one fecal test for *S. stercoralis*: three from sub-Saharan Africa, and six from Asia (Table 4). The prevalence of strongyloidiasis, defined as a positive serology and at least one positive fecal test, was 1.3% (95% CI 0.6%—2.5%).

**Table 4:** *Strongyloides stercoralis* results

	Region				p-value*
	Overall	Sub-Saharan Africa	Asia	North Africa	
Serology					< 0.001
Individuals screened by serology n	671	351	204	82	
Negative n (%)	623 (92.8%)	337 (96.0%)	204 (86.4%)	82 (97.6%)	
Positive n (%)	48 (7.2%)	14 (4.0%)	32 (13.6%)	2 (2.4%)	
Stool PCR					0.5
Individuals screened by stool PCR n	142	66	57	19	
Negative n (%)	138 (97.2%)	65 (98.5%)	55 (96.5%)	18 (94.7%)	
Positive n (%)	4 (2.8%)	1 (1.5%)	2 (3.5%)	1 (5.3%)	
Stool microscopy					0.7
Individuals screened by stool microscopy n	618	333	213	72	
Negative n (%)	615 (99.5%)	332 (99.7%)	211 (99.1%)	72 (100.0%)	
Positive n (%)	3 (0.5%)	1 (0.3%)	2 (0.9%)	0	
APC					> 0.9
Individuals screened by APC n	30	11	18	1	
Negative n (%)	21 (70.0%)	8 (72.7%)	12 (66.7%)	1 (100.0%)	
Positive n (%)	9 (30.0%)	3 (27.3%)	6 (33.3%)	0	
Missing n	644	341	220	83	

^*^Pearson’s Chi-squared test; Fisher’s exact test

Similarly, different assays were used for the screening of schistosomiasis, including serology for all forms of infection and diagnostics on stool and urine, targeting intestinal and urinary schistosomiasis, respectively (Table 5). Overall, 50 out of 388 participants was positive to at least one fecal or stool test, all from sub-Saharan Africa. Thus, the prevalence of schistosomiasis, defined as a positive serology and at least one positive fecal or urinary test, was 12.9% (95% CI 9.7%—16.6%).

**Table 5:** *Schistosoma* spp. results

	Region			p-value*
	Overall	Sub-Saharan Africa	North Africa	
Serology				0.084
Individuals screened by serology n	386	350	36	
Negative n (%)	183 (47.4%)	161 (46.0%)	22 (61.1%)	
Positive n (%)	203 (52.6%)	189 (54.0%)	14 (38.9%)	
Stool PCR				0.5
Individuals screened by stool PCR n	75	66	9	
Negative n (%)	69 (92.0%)	61 (92.4%)	8 (88.9%)	
Positive n (%)	6 (8.0%)	5 (7.6%)	1 (11.1%)	
Stool microscopy				0.2
Individuals screened by stool microscopy n	364	333	31	
Negative n (%)	336 (92.3%)	305 (91.6%)	31 (100.0%)	
Positive n (%)	28 (7.7%)	28 (8.4%)	0	
Urine PCR				
Individuals screened by urine PCR n	19	19	0	
Negative n (%)	19 (100.0%)	19 (100.0%)	0	
Positive n (%)	0 (0.0%)	0 (0.0%)	0	
Urine microscopy				0.2
Individuals screened by urine microscopy n	355	322	33	
Negative n (%)	335 (94.4%)	302 (93.8%)	33 (100.0%)	
Positive n (%)	20 (5.6%)	20 (6.2%)	0	
Missing n	81	30	51	

^*^Pearson’s Chi-squared test; Fisher’s exact test

Further, 332 individuals from sub-Saharan Africa were tested with the pan-filaria serology. Twenty-three individuals out of the 332 (6.9%) were positive, so were tested for microfilaremia. The latter was positive in six (26.1%) out of the 23 serology-positive individuals, permitting the diagnosis of two *Loa loa* and four *Mansonella perstans* cases. The prevalence of filariasis, defined by a positive serology and the presence of microfilaremia, was 1.8% (95% CI 0.7%—3.9%).

### Eosinophilia

The median eosinophil count was 200/µL (IQR 100–300) in the whole cohort, including 21% subjects (n = 141) with an eosinophil count ≥ 400/µL. Median eosinophil count in individuals from sub-Saharan Africa was 200 (IQR 100–300). For Asia, median eosinophil count was 200 (IQR 100–400). Median eosinophil count of people from North Africa was 100 (IQR 100–300).

As for eosinophilia by geographical region of origin, it was present in 64 of 351 (18.2%) individuals from sub-Saharan Africa (median eosinophil count 600 (IQR: 500–900)), 64 of 238 (26.9%) individuals from Asia (median eosinophil count 500 (IQR 400–800)), 13 of 84 (15.5%) subjects from North Africa (median eosinophil count 500 (IQR: 400–600)).

The multivariable model for sub-Saharan Africa found a significant association between eosinophilia and schistosomiasis (OR 4.31, 95%CI 2.10–8.84, p < 0.001).

For Asia, eosinophilia was significantly associated with strongyloidiasis (OR 8.11, 95%CI 1.44–82.6, p = 0.017) and with hookworm (OR 29.5, 95%CI 3.26–3,891, p < 0.001). As regards the sub-group of individuals from North Africa, models were not possible due to the extremely low frequency of parasitic infections diagnosed. Although *Ascaris lumbricoides* was included among the helminths screened through stool microscopy, it was not included in the multivariable analysis due to the very low number of positive cases.

## Discussion

The first notable finding from our study is the significant shift in the geographical origin of migrants. The proportion of migrants from Asia has increased to 35.3%, compared to 21% in our previous study (p < 0.001) [8]. This shift aligns with global migration trends and the most recent Italian estimates [1, 2]. Such a change in the geographical origin of migrants should be considered when adapting guidelines and recommendations for screening both infectious and non-infectious diseases to the evolving epidemiological landscape.

In this context, our study offers an updated overview of the prevalence of infectious diseases among recently arrived migrants in Italy, nine years after our previous work in the same setting [8]. The findings further confirm the relevance of infectious diseases in this population, with significant differences observed based on migrants' geographical origin.

HIV prevalence among migrants in this study was relatively low, with an overall rate of 1.5%, which is comparable to the prevalence observed in our previous study (1.3%) [8]. Similarly, there was a notable geographic disparity, with the majority of cases being observed among migrants from sub-Saharan Africa (90%). This is consistent with ECDC estimates as well as previous studies showing the highest HIV prevalence rates in sub-Saharan Africa, highlighting the continued need for comprehensive HIV screening and awareness programs targeting individuals from that geographical area [9, 10]. Although HIV prevalence was lower among migrants from Asia and North Africa in our cohort, we acknowledge that early diagnosis remains a public health priority. Therefore, our findings support the continued implementation of universal HIV screening among newly arrived migrants, irrespective of geographic origin.

Regarding HBV infection, 6.1% of participants were positive for HBsAg. Of note, HBsAg positivity in our study was lower than both our previous work (i.e. 11.6%) and similar studies on migrant populations [11]. Additionally, 55.1% of individuals had negative HBV serology, indicating a substantial proportion of susceptible individuals who could benefit from vaccination programs. The remaining 44.9% included both individuals with chronic HBV infection (6.1%) and those with serological evidence of past infection or immunity (i.e. anti-HBs and/or anti-HBc IgG positive, 38.8%). In particular, 75.9% of Asian migrants and 79.8% of North African migrants were eligible for HBV vaccination, emphasizing the need for tailored immunization strategies. Comparatively, the prevalence of chronic HBV infection (HBsAg positivity) in the general Italian population is approximately 0.8% [12]. The higher rate in our cohort, particularly among migrants from sub-Saharan Africa, likely reflects lower vaccination coverage and later adoption of universal vaccination in countries of origin. These findings support the importance of targeted screening and vaccination strategies aimed at newly arrived migrant populations to address health disparities.

Although the overall prevalence of HCV in our cohort was low (0.8%), it remains noteworthy, particularly among individuals from Pakistan. Only one of the five individuals with positive serology had detectable HCV-RNA, highlighting the importance of reflex testing for accurate diagnosis of active infection. This figure is comparable to or slightly lower than recent national estimates for the Italian general population, with anti-HCV antibody prevalence ranging around 1% and a viremic prevalence of approximately 0.66% [13]. These findings suggest that newly arrived migrants may not represent a significantly higher HCV burden compared to the host population; nonetheless, routine screening remains important to ensure early diagnosis and linkage to care.

Syphilis was detected in 2% of the migrants, which is consistent with findings from other studies that have addressed sexual health within migrant populations [14]. The prevalence was particularly high among sub-Saharan African migrants (3.5%) and relatively low in migrants from Asia and North Africa. In our previous study, 4.5% of participants from sub-Saharan Africa and 1.0% of those from Asia tested positive. This emphasizes the need for continued vigilance in screening for sexually transmitted infections, particularly in populations with higher rates of sexual risk behaviors and prior exposure in endemic regions.

Tuberculosis emerged as one of the most concerning findings in our study. The prevalence of TBI was 24.5%, while TB disease was diagnosed in 1.2% of participants, including both pulmonary and extrapulmonary forms. These results underscore the importance of comprehensive diagnostic strategies for TB among newly arrived migrants. The rate of TBI is consistent with epidemiological data from high-burden regions such as sub-Saharan Africa and Asia [15–18], from which most of our cohort originated. These findings reinforce the need to maintain systematic TB screening upon arrival in Europe and to initiate preventive treatment for TBI, which is essential to reduce the risk of disease reactivation and subsequent transmission within host countries.

A particularly relevant aspect of our study concerns helminthic infections. We observed an overall proportion of 12.3% for at least one helminthic infection diagnosed through a positive stool test and/or urine test, with clear differences by geographical region.

The prevalence of strongyloidiasis was 1.3%, which is consistent with the stool-based prevalence reported by Asundi et al. (1.8%) [19]. Our study also highlights a significant seroprevalence of strongyloidiasis (7.2%), with seropositivity markedly higher among Asian migrants (13.6%). Notably, our findings are lower than the pooled seroprevalence of 12.2% reported by Asundi et al. [19]. It should be noted that different serological assays have a wide range of sensitivity and specificity values, with most concerns relating the potential cross-reactivity with other nematodes [19, 20]. However, due to the potential development of severe or disseminated infection in cases of immunocompromise, treatment of individuals who are only positive for serology is considered justified. Therefore, in this setting, lower specificity is not regarded as problematic as lower sensitivity.

The prevalence of schistosomiasis was 12.9%, with all cases, as expected, found among migrants from sub-Saharan Africa. It is worth noting that our findings are higher than the stool-based prevalence of 0.95% and the urine-based prevalence of 6.8% reported by Asundi et al. [19]. Also, the seroprevalence for *Schistosoma* spp. in our cohort was higher (52.6%) than that reported by Asundi et al. (18.4%), likely reflecting the low specificity of the test and the need for alternative diagnostic approaches for schistosomiasis, similarly to strongyloidiasis [19].

Filariasis remains an underdiagnosed parasitic disease in migrant populations, with limited data available in non-endemic settings [21]. Data on the prevalence of filariasis among migrants in Europe remain scarce, and most available epidemiological evidence comes from studies conducted in endemic regions of Africa [22, 23]. This knowledge gap complicates the development of targeted screening strategies in migrant populations, particularly given the clinical implications of filarial infections. Loiasis has recently been associated with increased mortality in cases with a high microfilarial burden, with eyeworm and Calabar swellings as characteristic clinical features; however, the disease can also present with atypical, non-specific symptoms [24]. While infection with *M. perstans* is generally considered less severe than other filarial infections, it can still lead to long-term symptoms and complications in certain individuals, such as abdominal pain and dermatitis [25]. Moreover, the stool tests for other parasitic diseases, including hookworm and *T. trichiura*, revealed significant rates of infection, particularly among migrants from Asia.

These findings confirm that routine screening for helminths is key a component of migrant health assessments, especially since these infections are often asymptomatic in the early stages but can lead to severe health consequences if untreated [3, 4, 8]. The cost-effectiveness and ease of treatment for these parasitic infections further emphasize the importance of including them in national screening protocols for newly arrived migrants. Similar findings have been reported by the REDIVI network in Spain, which analyzed trends in imported infections among more than 14,000 migrants and travelers over the past decade, further supporting the value of structured screening approaches in Europe [26]. Moreover, the heterogeneity in infection prevalence by region of origin supports the need for geographically-tailored screening strategies. As migration patterns evolve over time, adopting a flexible, route- and country-based approach could improve the effectiveness and efficiency of screening programs. While our study did not formally propose such a framework, the data provided may contribute to its future development.

Eosinophilia was present in 18.3% of the screened migrants and was significantly associated with helminthic infections. This association was especially pronounced among migrants from sub-Saharan Africa and Asia. Our analysis confirmed that *S. stercoralis* and *Schistosoma* spp. were the most frequently associated parasites, in line with previous studies [27, 28]. Specifically, eosinophilia was observed in 18.2% of African migrants and 26.9% of Asian migrants, with the condition predominantly linked to schistosomiasis in African migrants and strongyloidiasis in Asian migrants. The sensitivity of eosinophilia as a marker for helminthiasis is well recognized, but its specificity remains low, as it can be influenced by non-infectious conditions such as allergies and autoimmune diseases [27, 29]. Remarkably, our findings suggest that eosinophilia alone is insufficient to rule out helminthic infections. Among *S. stercoralis* cases, 2/9 (22.2%) of infected individuals did not present eosinophilia, emphasizing the need for systematic screening. Similarly, 27/50 (54.0%) patients with *Schistosoma* spp. infections did not have eosinophilia, suggesting that chronic infections may not always trigger a sustained eosinophilic response [30]. These results highlight the importance of a combined diagnostic approach, integrating eosinophilia assessment with direct parasitological methods, serology, and molecular techniques to improve case detection.

One of the main limitations of our study is its retrospective nature, which may have affected the quality of data collection. A comparison of prevalence with data from previous studies is challenging due to differences in the definitions of helminthic infections and the use of various diagnostic tests for screening (e.g., different serological assays for *S. stercoralis* and *Schistosoma* spp.). Additionally, the collinearity between eosinophilia and the presence of certain parasites posed a challenge in constructing robust multivariable models to assess risk factors, as reflected in the wide confidence intervals of the OR estimates. An additional limitation is that eosinophilia was defined solely based on the absolute eosinophil count, without considering the eosinophil percentage. This approach may have entailed an underestimation of eosinophilia in individuals with low total leukocyte counts, such as those from sub-Saharan Africa. Furthermore, data on VDRL titers and previous syphilis treatment were inconsistently recorded and therefore could not be included in the analysis.

Furthermore, our findings reflect the specific characteristics of the local migrant population, which may limit their applicability to other settings with different demographic and epidemiological profiles. Additionally, the distinction between asylum seekers and undocumented migrants was not addressed in the paper, and no analysis was performed to explore potential differences between these two categories.

Finally, with regard to TB classification, we did not distinguish between subclinical and symptomatic forms of TB [31], as this distinction was beyond the scope and focus of the present study.

## Conclusions

Our findings support the need for a tailored approach to screening for infectious diseases in migrants, adapting strategies based on geographical origin. The integration of effective screening protocols and expanded access to vaccination and early treatment could significantly improve both individual and public health outcomes.

Helminthic infections, particularly *S. stercoralis* and *Schistosoma* spp., remain highly prevalent, and eosinophilia alone is not a sufficient screening tool. Systematic, evidence-based screening programs are essential to ensure early detection and treatment, reducing the burden of infectious diseases in migrant populations and preventing long-term complications.

## Data Availability

The data underlying this article will be shared on reasonable request to the corresponding author.
